# Primary thyroid chondrosarcoma: a case report of an extremely rare malignancy

**DOI:** 10.1186/s43046-022-00138-z

**Published:** 2022-08-29

**Authors:** Shadi Awny, Mohammad Zuhdy, Omar Hamdy, Gehad Ahmad Saleh, Ahmed Hassan, Mohamed Abdelkhalek, Amir Mosaad, Mohamed T. Hafez, Sameh Roshdy, Ahmed Setit, Nirmeen Megahed

**Affiliations:** 1 Surgical Oncology Unit, Oncology Centre Mansoura University (OCMU), Mansoura, Egypt; 2 Radiology Department, Faculty of Medicine, Mansoura University, Mansoura, Egypt; 3 Pathology Department, Faculty of Medicine, Mansoura University, Mansoura, Egypt

**Keywords:** Thyroid cancer, Thyroid sarcomas, Thyroid chondrosarcoma, Case report

## Abstract

**Introduction:**

There are different types of malignant tumors that can affect the thyroid gland where differentiated thyroid carcinomas (papillary and follicular) are the most common representing nearly 90% of cases. Non-epithelial malignancies were also reported to affect the thyroid gland particularly lymphomas and sarcomas that were reported in literature to range from 0.01 to 1.5% of thyroid carcinoma. Herein, we present a case with primary thyroid chondrosarcoma, an extremely rare malignancy of the thyroid gland.

**Case presentation:**

We present a 79-year-old female patient complaining of hard thyroid swelling that was proved to be primary thyroid chondrosarcoma after histopathological assessment.

**Conclusion:**

Chondrosarcoma of the thyroid gland is extremely rare either in the primary or metastatic setting. Although the prognosis is bad, surgery is the main line of treatment after early prompt diagnosis.

## Introduction

Thyroid cancers are very common malignancies with female predominance. Papillary carcinoma is the commonest type. Differentiated thyroid carcinomas (papillary and follicular) represent about 90% of cases, while medullary carcinoma represents about 5%. Rare types include anaplastic carcinomas and lymphomas. Sarcomas can also arise within the thyroid gland with very low frequency. Chondrosarcomas are a group of heterogenous cartilaginous neoplasms that account for 20 to 25% of primary bone malignancy. They mostly metastasize through the hematogenous route to distant sites predominantly lungs followed by skin and soft tissues [1, 2]. Herein, we present a case with primary thyroid chondrosarcoma that was diagnosed in an elderly female after thorough histopathological examination and immune histochemical staining. This is an extremely rare malignancy of the thyroid gland with only few case reports available in literature.

## Case presentation

A 79-year-old woman without significant medical or surgical history was referred to our hospital complaining of a slowly growing anterior neck swelling. She stated that this swelling started to appear in her neck 6 months ago. The swelling was painless and gradually increasing in size. She refused to seek medical advice throughout those months till she started to feel some respiratory discomfort.

**Fig. 1:**
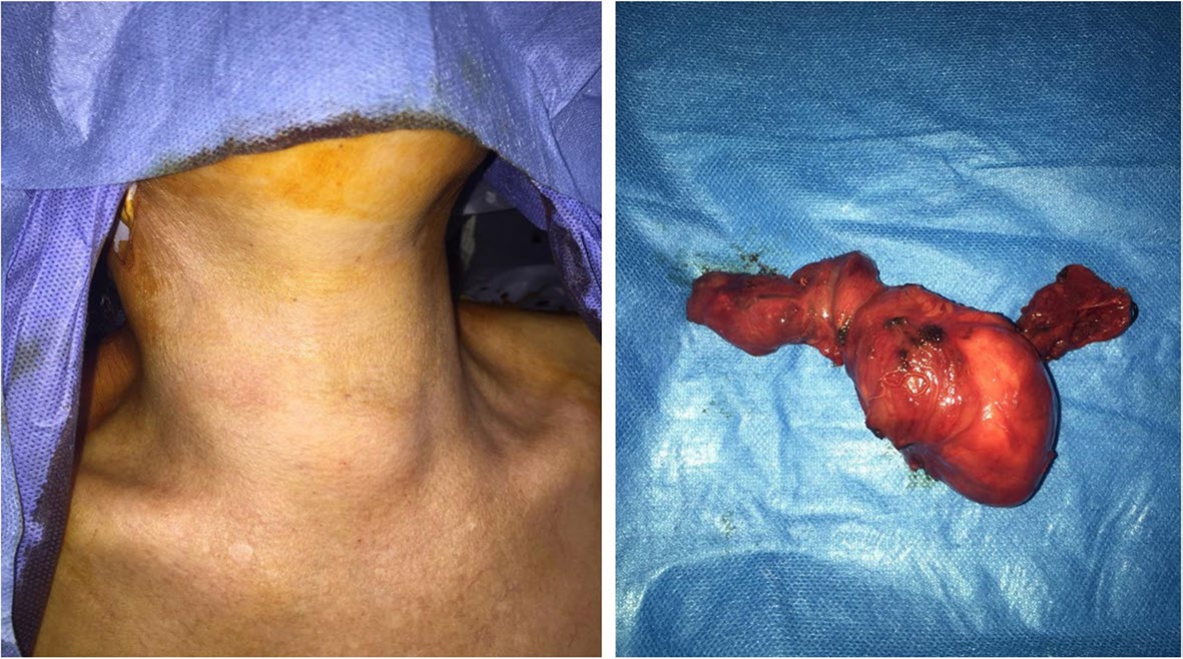
**A** Preoperative photo showing the thyroid swelling with undefined lower border. **B** Postoperative photo of the resected thyroid gland showing replaced right lobe by large hard nodule

Clinical examination revealed a stony hard neck swelling that moved up and down with deglutition, mostly of thyroid origin. The swelling was not tender and its lower border could not be felt by palpation, denoting the possibility of retrosternal extension (Fig. 1A).

Neck ultrasonography showed a nodule in the lower pole of the right thyroid lobe. This nodule was hypoechoic with internal vascularity, microcalcification, and cystic degeneration measuring about 3–4 cm. It was categorized as TIRADS 4b [3]. It extended down to the mediastinum with some enlarged cervical lymph nodes with preserved criteria.

**Fig. 2:**
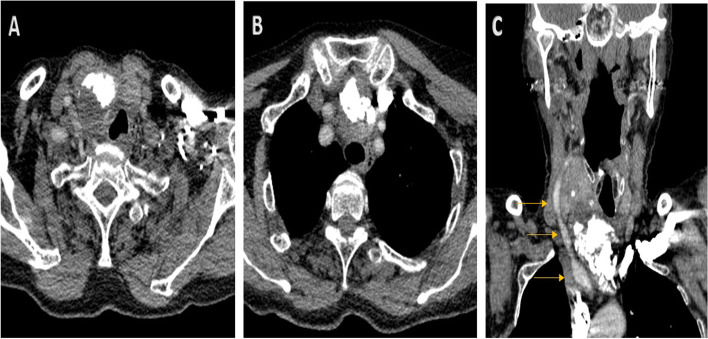
Post-contrast neck and upper chest CT axial (**A**, **B**) and coronal images (**C**). Heterogenous enhancing right thyroid lobe soft tissue mass with coarse calcification is seen displacing the airway to the contralateral side and extending down to the superior mediastinum abutting the aortic arch branches with clear fat plane in-between. It was seen also contacting the right CCA along its length without vascular invasion (arrows). No pathological cervical LNs

Computerized tomography scan with contrast of the neck and chest was done and revealed a soft tissue mass measuring 3.3×4.8 cm inseparable from the right thyroid lobe, contacting strap muscles anteriorly and prevertebral muscles posteriorly, compressing and displacing trachea to the left side, contacting the right carotid sheath with neither encasement nor infiltration. The mass was extending inferiorly to the mediastinum and left brachiocephalic arch and reaching the level of the fifth cervical vertebra (Fig. 2). Few nodules were seen in both lung fields; the largest was measuring 5 mm in the left anterior segment of the left lung for follow-up.

Thyroid function tests were of normal values and her vocal cords were freely mobile bilaterally.

FNAC was taken from the hard thyroid swelling. Smears prepared were moderately cellular showing occasional scattered groups of cells with a moderate degree of atypia and pleomorphism in RBCs background, categorized as Besthesda V [4].

The patient was prepared for surgery after a multidisciplinary discussion. Total thyroidectomy was done, visualizing both recurrent laryngeal nerves and preserving the parathyroid glands. The surgery was adopted via a fully cervical approach “Kocher’s collar incision” without sternotomy. The patient had a smooth postoperative course and was discharged 48 h after surgery.

The right lobe measured 8×8×4 cm and the left lobe measured 4×3×2 cm. Dissection of the right lobe revealed a tissue nodule measuring 6×5×4 cm. It had hard cut surface, whitish alternating with yellowish areas. The left lobe showed a nodular glistening brownish cut surface (Fig. 1B).

**Fig. 3:**
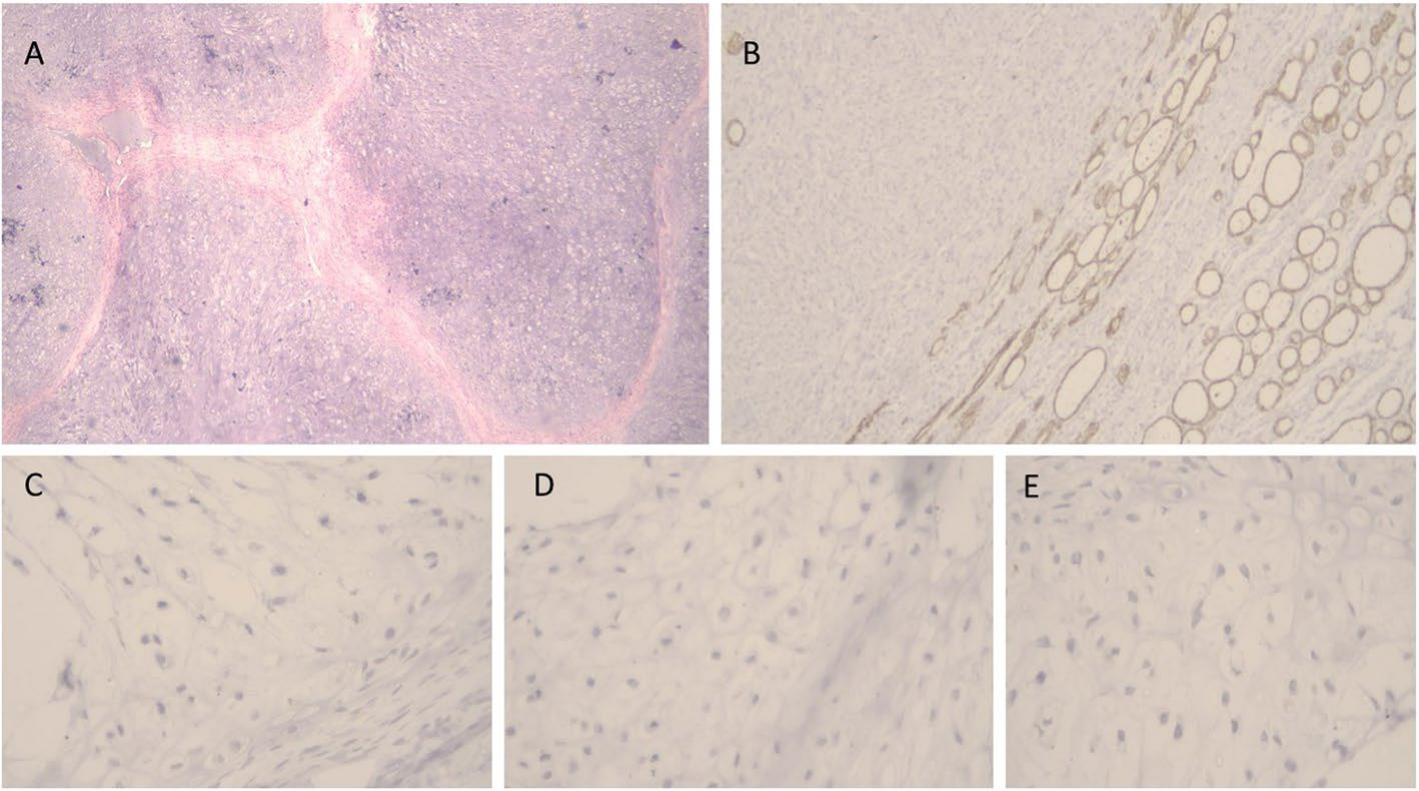
**A** Tumor formed of lobules of malignant chondrocytes embedded in a chondroid matrix and separated by fibrous septa, H&E 100×. **B** Tumor cells were negative for thyroglobulin in contrast to entrapped thyroid follicles which were positive for thyroglobulin 200×. **C** Tumor cells were negative for TTF-1, 400×. **D** Tumor cells were negative for PAX-8, 400×. **E** Tumor cells were negative for CK, 400×

Sections prepared from the right lobe revealed infiltration by well-circumscribed lobulated malignant tumoral proliferation formed of cartilaginous matrix with chondrocytes embedded in lacunae and showing hypercellularity, occasional binucleate nuclei and scarce mitosis. Foci of myxoid degeneration were also seen. No detected necrosis. Focal bone formation was detected. Sections from the left lobe and isthmus were free. Slides stained for cytokeratin, EMA, P63, TTF1, Pax8, and thyroglobulin were negative in the tumor cells (Fig. 3). Ki-67 was less than 5% which is against high ki-67 proliferation index usually seen in anaplastic thyroid carcinoma. Picture of low-grade chondrosarcoma of right thyroid lobe was the final pathologic diagnosis.

Comprehensive immunohistochemical studies showed that this tumor is not consistent with anaplastic thyroid carcinoma with chondrosarcomatous differentiation as the tumor cells were totally negative for TTF-1, thyroglobulin, and PAX-8 which are the major three markers used in immunohistochemical studies for the determination of thyroid origin. In addition, tumor cells were completely negative for panCK, CK7, CK20, EMA, and P63. Lastly Ki-67 was less than 5% which is against high ki-67 proliferation index usually seen in anaplastic thyroid carcinoma. These findings were all supportive for the diagnosis of low grade chondrosarcoma

The possibility of metastatic chondrosarcoma to the thyroid gland was ruled out by a computerized scan of the whole body’s skeleton that did not detect any primary tumor. MDT discussion agreed for active surveillance of the patient on levothyroxine replacement therapy.

Six months postoperative, the patient was in a good general condition; her follow-up CT examination revealed clear operative bed, with calcified paratracheal nodes measuring about 8×10 mm. Bilateral multiple calcified pulmonary nodules were also detected; the largest was in the left upper lung lobe measuring 8×7 mm possibly metastatic regarding the patient’s history.

A computerized scan of the chest 18 months postoperative revealed a progressive course of the pulmonary nodules reaching a size of 25mm with moderate right-sided pleural effusion that was proved to be malignant by cytological evaluation. At that time the patient’s performance declined to ECOG 3. That is why the multi-disciplinary tumor board recommended best supportive care and pain therapy measures.

## Discussion

Thyroid carcinoma is the most common endocrine malignancy and the fifth most common among females [1]. There are various pathological subtypes including the most common differentiated types (papillary, follicular, and Hurthle cell carcinoma), the medullary, and the anaplastic thyroid cancer [5].

Non-epithelial malignancies were also reported to affect the thyroid gland particularly lymphomas and sarcomas. Their incidence is very low reaching 1.5–5% in lymphomas while in sarcomas, the incidence of primary thyroid sarcomas ranged from 0.01 to 1.5% [6]. Chondrosarcoma is reported to arise from extraskeletal sites but it is rarely reported to affect solid parenchymatous organs [7]. We herein report a case of primary thyroid chondrosarcoma.

Chondrosarcomas are slowly growing invasive tumors that comprise nearly 11% of all primary malignant bone tumors. Nearly 12% of chondrosarcoma could affect the head and neck region [8]. The most common histological type of chondrosarcoma is the conventional type representing approximately 90% while the remaining 10% includes the myxoid, dedifferentiated, clear, and mesenchymal types. Conventional chondrosarcomas can be graded grade I, II, or III depending on nuclear size, differentiation, and cellular density [9]. Both primary and metastatic chondrosarcoma of the thyroid are rarely reported in literature with only a few cases previously reported [10].

In their systematic review, Surov et al. analyzed the clinical data about primary thyroid sarcomas in literature. They reported angiosarcoma, malignant hemangioendothelioma, and malignant fibrous histiocytoma to be the most common pathological types of primary thyroid sarcoma. On the other hand, primary thyroid chondrosarcoma was only reported once in literature when Abbas et al. reported a case of extraskeletal mesenchymal chondrosarcoma of the thyroid gland in a 13-year-old female [6, 7]. Interestingly, earlier reports about primary chondrosarcoma in elderly males were described by Jasonni et al., Tselini-Balafouta et al., and Haeri et al., while the most recent report of a 24-year-old Asian male with mesenchymal chondrosarcoma of the thyroid gland with uncertain primary was by Nachawi et al. in 2020 [10–13]. Thyroid metastases from chondrosarcoma were also rarely reported in literature. Prognosis was reported to be poor depending mainly on the primary tumor. Thyroidectomy was thought to improve the patients’ quality of life without an effect on survival [8, 14–16].

The American Thyroid Association published the guidelines on the management of thyroid nodules in 2015 and recommended fine needle aspiration cytology from any non-functioning thyroid nodule greater than 1 cm in diameter with intermediate and/or high suspicion for malignancy [17]. That is why FNAC was performed in our case and it revealed high suspicion for malignancy categorized as Bethesda V although it failed to reach the diagnosis of chondrosarcoma. The available evidence from literature recommends core needle biopsy over fine-needle aspiration in the diagnosis of bone and soft tissue sarcomas [18].

In the present case, the pathological diagnosis of low-grade conventional chondrosarcoma was confirmed after using immune histochemical stains on paraffin-stained sections of the thyroidectomy specimen. Tumor cells were negative for cytokeratin, S-100 & EMA, P63, TTF1, Pax8, and thyroglobulin. The possibility of metastatic chondrosarcoma was excluded due to the absence of any primary tumor by postoperative computerized tomography scan. Several theories explained the remote possibility of intrathyroid metastasis despite its rich vascularity. The most accepted of them are the carcinostatic effect of iodine and the concentration of thyroid hormones and antitumor factors within the thyroid gland [8]. Pathological diagnosis could be confused with the rare small cell variant of medullary thyroid carcinoma but cartilage would be absent and CEA would be negative. In addition, anaplastic thyroid carcinoma might contain cartilage, hemangiopericytoma-like areas, and osteoclast-like giant cells but the tumor cells would be pleomorphic and cytokeratin stained [7] which was not encountered in our case.

Unfortunately, there are no available general recommendations for the treatment of thyroid sarcomas unlike thyroid cancer [6]. Complete surgical excision is the mainstay of treatment of chondrosarcoma. Other available lines of treatment include chemotherapy, radiation, and targeted therapy. Thanks to poor vascularity, extracellular matrix, and low percentage of cellular proliferation, chondrosarcomas are considered resistant to both radiation and chemotherapy [19]. In the present case, the patient developed progressive pulmonary metastasis after curative thyroidectomy. The multidisciplinary tumor board recommended best supportive care and pain therapy because of the poor performance status of the patient.

## Conclusion

Chondrosarcoma of the thyroid gland is extremely rare either in the primary or metastatic setting. Although the prognosis is bad, surgery is the main line of treatment after early prompt diagnosis.

## Data Availability

The data for current study is available from corresponding author at reasonable request.

## Abbreviations

TIRADS: Thyroid Imaging and Radiological Data System
FNAC: Fine needle aspiration cytology
RBCs: Red blood cells
CT: Computed tomography
EMA: Epithelial membrane antigen
P63: Tumor protein 63
TTF1: Thyroid transcription factor 1
Pax8: Paired-box gene 8
MDT: Multidisciplinary team
ECOG: Eastern Cooperative Oncology Group
CEA: Carcinoembryonic antigen
